# Sulphonamides incorporating 1,3,5-triazine structural motifs show antioxidant, acetylcholinesterase, butyrylcholinesterase, and tyrosinase inhibitory profile

**DOI:** 10.1080/14756366.2019.1707196

**Published:** 2020-01-03

**Authors:** Nabih Lolak, Mehmet Boga, Muhammed Tuneg, Gulcin Karakoc, Suleyman Akocak, Claudiu T. Supuran

**Affiliations:** a Department of Pharmaceutical Chemistry, Faculty of Pharmacy, Adiyaman University, Adiyaman, Turkey; b Department of Analytical Chemistry, Faculty of Pharmacy, Dicle University, Diyarbakir, Turkey; c NEUROFARBA Department, Sezione di Scienze Farmaceutiche, Università degli Studi di Firenze, Florence, Italy

**Keywords:** Benzenesulfonamides, 1,3,5-triazine, enzyme inhibition, Alzheimer’s disease, tyrosinase

## Abstract

A series of 16 novel benzenesulfonamides incorporating 1,3,5-triazine moieties substituted with aromatic amines, dimethylamine, morpholine and piperidine were investigated. These compounds were assayed for antioxidant properties by using 1,1-diphenyl-2-picrylhydrazyl (DPPH) radical scavenging assay, 2,2`-azino-bis(3-ethylbenzothiazoline-6-sulfonic acid (ABTS) radical decolarisation assay and metal chelating methods. They were also investigated as inhibitors of acetylcholinesterase (AChE), butyrylcholinesterase (BChE) and tyrosinase, which are associated with several diseases such as Alzheimer, Parkinson and pigmentation disorders. These benzenesulfonamides showed moderate DPPH radical scavenging and metal chelating activity, and low ABTS cation radical scavenging activity. Compounds **2 b**, **3d** and **3 h** showed inhibitory potency against AChE with % inhibition values of >90. BChE was also effectively inhibited by most of the synthesised compounds with >90% inhibition potency. Tyrosinase was less inhibited by these compounds.

## 1. Introduction

The 1,3,5-triazine scaffold, also known as s-triazine, and its derivatives have a wide range of applications due to broad biological activities including antiviral, antibacterial, anti-inflammatory, anti-HIV and more recently anti-cancer activity^1–4^. Specifically, sulphonamides incorporating 1,3,5-triazine moieties were extensively studied as a potent and selective carbonic anhydrase inhibitors^5–10^. Some of these compounds showed the best selectivity ratio for tumour over-expressed membrane-bound carbonic anhydrase isoform IX (hCA IX) over the off-target isoform II (hCA II), between 166 to 706 fold^5–7^. More recently, ureido benzenesulfonamides incorporating 1,3,5-triazine motifs were investigated as a potent class of inhibitors of the cancer related isoform human carbonic anhydrase IX (hCA IX) by our group^11^^,^^12^.

Alzheimer’s disease (AD), a progressive neurodegenerative disorder of the brain, is characterised by cognitive dysfunction, memory decrease, speech impairment and dementia, which is the leading cause of disability in elderly people in the world^13–15^. It affects about 50 million people worldwide and this number might triplicate to 152 million by 2050 (World Alzheimer report)^16^, as life expectancy is increasing worldwide. Acetylcholine (ACh), a neurotransmitter responsible for the conduction of electrical impulses from one nerve to another, is one of the main biochemical alteration in the brain of patients with Alzheimer^16–18^. The level of ACh decreases due to its hydrolysis by acetylcholinesterase (AChE), which is a terminator enzyme of nerve impulse transmission. Butyrylcholinesterase (BChE) is a non-specific cholinesterase enzyme that hydrolyses many different choline-based esters^19^^,^^20^. Thus, AChE and BChE inhibition are being considered as a one of the most possible approaches for the treatment of AD^21^^,^^22^. There are several cholinesterase inhibitors used clinically for the treatment of AD such as, donepezil, tacrine, rivastigmine and galanthamine. However, these drugs have a limited efficacy, are toxic and have unfavourable side effects such as dizziness, hepatotoxicity, diarrhoea, vomiting and nausea^21^^,^^22^. For these reasons, there is an urgent need for more potent and highly efficient cholinesterase inhibitors for the management of AD.

It has recently been documented that 1,3,5-triazine derivatives, specifically sulphonamide substituted ones, may show interesting biological properties as enzyme inhibitors^5–12^. To the best of our knowledge, there are no literature reports on the antioxidant, anticholinesterase and tyrosinase activities of 1,3,5-triazines containing sulphonamides. Therefore, in this study, we aimed to evaluate the antioxidant, anticholinesterase and tyrosinase activities of novel sulphonamides incorporating 1,3,5-triazine moieties.

## 2. Materials and methods

### 2.1. Chemistry

All chemicals and anhydrous solvents were purchased from Sigma-Aldrich, Merck, Alfa Aesar and TCI and used without further purification. FT-IR spectra were obtained by using Perkin Elmer Spectrum 100 FT-IR spectrometer. Nuclear Magnetic Resonance (^1^H-NMR and ^13 ^C-NMR) spectra of compounds were recorded using a Bruker Advance III 300 MHz spectrometer in DMSO-*d_6_* and TMS as an internal standard operating at 300 MHz for ^1^H-NMR and 75 MHz for ^13 ^C-NMR. Thin-layer chromatography (TLC) was carried out on Merck silica gel 60 F_254_ plates.

#### 2.1.1. General procedure for the synthesis of compounds 2(a–d)

At 0–5 °C, a 10 mmol solution of R_1_ (aromatic amine derivatives, -4 F, -4MeO, -3,4diCl, -3NO_2_) was added to 5 mmol of compound **1** in DMF under stirring. After complete addition, the mixture was allowed to warm to room temperature for 1 h, after that the reaction mixture was heated to 30–40 °C for 6–8 h. Then, the product was filtered off, washed with water and dried under vacuum at 40 °C. The obtained final pure products were fully characterised by FT-IR, ^1^H-NMR, ^13 ^C-NMR, and melting points.

##### 4-((4-chloro-6-((4-fluorophenyl)amino)-1,3,5-triazin-2-yl)amino)benzenesulfonamide (2a)

Yield: 75%; Colour: white solid; m.p.: 262–265 °C; FT-IR (cm^−1^): 3418, 3309, 3248, 1617, 1496 (asymmetric), 1322, 1157 (symmetric) (S = O); ^1^H-NMR (DMSO-*d_6_*, 300 MHz, δ ppm): 7.92 (d, 2H, *J* = 6.3, Ar-H), 7.85 (d, 2H, *J* = 6.3, Ar-H), 7.76 (d, 2H, *J* = 6.6, Ar-H), 7. 55 (d, 2H, *J* = 6.3, Ar-H), 7.38 (s, 2H, -SO_2_NH_2_): ^13 ^C-NMR (DMSO-*d_6_*, 75 MHz, δ ppm): 169.8 (C-Cl), 167.2, 165.5 (C_triaz_-N), 157.4 (C-F), 143.7, 135.4, 130.9, 130.1, 120.7, 116.4, 113.7;

##### 4-((4-chloro-6-((4-methoxyphenyl)amino)-1,3,5-triazin-2-yl)amino)benzenesulfonamide (2 b)

Yield: 68%; Colour: white solid; FT-IR (cm^−1^): 3447, 3316, 3255, 1623, 1505 (asymmetric), 1329, 1162 (symmetric) (S = O); ^1^H-NMR (DMSO-d_6_, 300 MHz, δ ppm): 7.89 (d, 2H, *J* = 6.3, Ar-H), 7.81 (d, 2H, *J* = 6.3, Ar-H), 7.74 (d, 2H, *J* = 6.6, Ar-H), 7. 45 (d, 2H, *J* = 6.3, Ar-H), 7.33 (s, 2H, -SO_2_NH_2_), 3.85 (s, 3H, -OCH_3_): ^13 ^C-NMR (DMSO-*d_6_*, 75 MHz, δ ppm): 169.5 (C-Cl), 167.1, 165.2 (C_triaz_-N), 156.3, 143.2, 135.8, 131.4, 130.3, 120.4, 116.2, 113.3, 56.5;

##### 4-((4-chloro-6-((3,4-dichlorophenyl)amino)-1,3,5-triazin-2-yl)amino)benzenesulfonamide (2c)

Yield: 55%; Colour: white solid; m.p.: 193–196 °C; FT-IR (cm^−1^): 3430, 3306, 3262, 1620, 1508 (asymmetric), 1336, 1160 (symmetric) (S = O); ^1^H-NMR (DMSO-d_6_, 300 MHz, δ ppm): 8.06 (d, 2H, *J* = 6.3, Ar-H), 7.92 (d, 2H, *J* = 6.3, Ar-H), 7.60 (s, H, Ar-H), 7. 58 (d, 2H, *J* = 6.3, Ar-H), 7.45 (s, 2H, -SO_2_NH_2_): ^13 ^C-NMR (DMSO-d_6_, 75 MHz, δ ppm): 170.2, 167.5, 165.7, 164.1, 157.2, 150.4, 142.9, 136.3, 130.6, 129.9, 126.4, 120.6, 118.1;

##### 4-((4-chloro-6-((3-nitrophenyl)amino)-1,3,5-triazin-2-yl)amino)benzenesulfonamide (2d)

Yield: 68%; Colour: white solid; FT-IR (cm^−1^): 3337, 3272, 3132, 1614, 1490 (asymmetric), 1350, 1156 (symmetric) (S = O); ^1^H-NMR (DMSO-d_6_, 300 MHz, δ ppm): 7.95 (d, 2H, *J* = 6.0, Ar-H), 7.89 (d, 2H, *J* = 6.0, Ar-H), 7.58 (s, H, Ar-H), 7. 56 (m, 3H, Ar-H), 7.42 (s, 2H, -SO_2_NH_2_): ^13 ^C-NMR (DMSO-*d_6_*, 75 MHz, δ ppm): 169.8, 167.2, 165.1, 163.8, 157.0, 150.8, 142.3, 135.9, 130.2, 129.5, 125.8, 120.1, 117.5;

#### 2.1.2. General procedure for the synthesis of compounds 3(a-l)

Under stirring, a 2 mmol solution of R_2_-H (dimethyl amine, morpholine and piperidine) was added to 1 mmol of **2(a-d)** in DMF at room temperature for 1 h. Then, the reaction temperature was raised to 90 °C for 5 h. After cooling to room temperature, the mixture was filtered and the precipitate was washed with water and dried at 50 °C. The obtained final pure products **3(a-o)** were fully characterised by FT-IR, ^1^H-NMR, ^13 ^C-NMR, and melting points.

##### 4-((4-(dimethylamino)-6-((4-fluorophenyl)amino)-1,3,5-triazin-2-yl)amino) benzenesulfonamide (3a)

Yield: 77%; Colour: white solid; m.p.: 222–225 °C; FT-IR (cm^−1^): 3366, 3325, 3262, 1625, 1485 (asymmetric), 1337, 1159 (symmetric) (S = O); ^1^H-NMR (DMSO-d_6_, 300 MHz, δ ppm): 8.05 (d, 2H, *J* = 6.3 Hz, Ar-H), 7.91 (d, 2H, *J* = 6.3 Hz, Ar-H), 7.72 (d, 2H, *J* = 6.6 Hz, Ar-H), 7. 58 (d, 2H, *J* = 6.6 Hz, Ar-H), 7.49 (s, 2H, -SO_2_NH_2_), 3.05 (s, 6H, -CH_3_): ^13 ^C-NMR (DMSO-*d_6_*, 75 MHz, δ ppm): 167.2, 165.6, 164.5, 157.2, 143.5, 136.4, 130.8, 129.3, 120.9, 117.2, 36.8;

##### 4-((4-((4-fluorophenyl)amino)-6-morpholino-1,3,5-triazin-2-yl)amino)benzenesulfonamide (3 b)

Yield: 83%; Colour: white solid; m.p.: 249–252 °C; FT-IR (cm^−1^): 3334, 3263, 3202, 1605, 1484 (asymmetric), 1356, 1152 (symmetric) (S = O); ^1^H-NMR (DMSO-*d_6_*, 300 MHz, δ ppm): 8.01 (d, 2H, *J* = 6.6 Hz, Ar-H), 7.89 (d, 2H, *J* = 6.3 Hz, Ar-H), 7.70 (d, 2H, *J* = 6.6 Hz, Ar-H), 7. 55 (d, 2H, *J* = 6.6 Hz, Ar-H), 7.46 (s, 2H, -SO_2_NH_2_), 3.80–3.42 (m, 8H, morpholine): ^13 ^C-NMR (DMSO-*d_6_*, 75 MHz, δ ppm): 166.9, 165.3, 164.2, 157.5, 143.1, 136.6, 130.3, 129.5, 120.7, 117.6, 65.4, 42.5;

##### 4-((4-((4-fluorophenyl)amino)-6-(piperidin-1-yl)-1,3,5-triazin-2-yl)amino) benzenesulfonamide (3c)

Yield: 85%; Colour: white solid; m.p.: 228–230 °C; FT-IR (cm^−1^): 3319, 3260, 1603, 1494 (asymmetric), 1336, 1151 (symmetric) (S = O); ^1^H-NMR (DMSO-*d_6_*, 300 MHz, δ ppm): 8.04 (d, 2H, *J* = 6.6 Hz, Ar-H), 7.88 (d, 2H, *J* = 6.3 Hz, Ar-H), 7.72 (d, 2H, *J* = 6.6 Hz, Ar-H), 7. 56 (d, 2H, *J* = 6.6 Hz, Ar-H), 7.47 (s, 2H, -SO_2_NH_2_), 3.43–3.21 (m, 4H, piperidine), 1.75–1.45 (m, 6H, piperidine): ^13 ^C-NMR (DMSO-*d_6_*, 75 MHz, δ ppm): 166.7, 165.4, 164.1, 157.6, 143.0, 136.8, 130.5, 129.4, 120.8, 117.3, 42.7, 25.4, 24.2;

##### 4-((4-(dimethylamino)-6-((4-methoxyphenyl)amino)-1,3,5-triazin-2-yl)amino) benzenesulfonamide (3d)

Yield: 77%; Colour: cream solid; FT-IR (cm^−1^): 3447, 3310, 3265, 1613, 1495 (asymmetric), 1325, 1160 (symmetric) (S = O); ^1^H-NMR (DMSO-*d_6_*, 300 MHz, δ ppm): 7.87 (d, 2H, *J* = 6.6, Ar-H), 7.79 (d, 2H, *J* = 6.3, Ar-H), 7.70 (d, 2H, *J* = 6.6, Ar-H), 7. 46 (d, 2H, *J* = 6.3, Ar-H), 7.38 (s, 2H, -SO_2_NH_2_), 3.87 (s, 3H, -OCH_3_), 3.03 (s, 6H, -CH_3_): ^13 ^C-NMR (DMSO-*d_6_*, 75 MHz, δ ppm): 169.3, 167.0, 165.4, 156.9, 143.8, 136.2, 131.2, 129.9, 120.6, 116.9, 56.8, 36.9;

##### 4-((4-((4-methoxyphenyl)amino)-6-morpholino-1,3,5-triazin-2-yl)amino) benzenesulfonamide (3e)

Yield: 80%; Colour: cream solid, m.p.: 209–212 °C; FT-IR (cm^−1^): 3437, 3305, 3255, 1610, 1502 (asymmetric), 1328, 1159 (symmetric) (S = O); ^1^H-NMR (DMSO-*d_6_*, 300 MHz, δ ppm): 7.90 (d, 2H, *J* = 6.3, Ar-H), 7.82 (d, 2H, *J* = 6.0, Ar-H), 7.73 (d, 2H, *J* = 6.6, Ar-H), 7. 49 (d, 2H, *J* = 6.3, Ar-H), 7.39 (s, 2H, -SO_2_NH_2_), 3.86 (s, 3H, -OCH_3_), 3.79–3.43 (m, 8H, morpholine): ^13 ^C-NMR (DMSO-*d_6_*, 75 MHz, δ ppm): 169.7, 167.3, 165.2, 157.3, 143.2, 136.4, 131.0, 129.8, 120.2, 116.6, 65.3, 56.7, 42.2;

##### 4-((4-((4-methoxyphenyl)amino)-6-(piperidin-1-yl)-1,3,5-triazin-2-yl)amino) benzenesulfonamide (3f)

Yield: 82%; Colour: cream solid, m.p.: 252–254 °C; FT-IR (cm^−1^): 3425, 3312, 3248, 1605, 1501 (asymmetric), 1332, 1160 (symmetric) (S = O); ^1^H-NMR (DMSO-*d_6_*, 300 MHz, δ ppm): 7.88 (d, 2H, *J* = 6.3, Ar-H), 7.80 (d, 2H, *J* = 6.0, Ar-H), 7.71 (d, 2H, *J* = 6.6, Ar-H), 7. 47 (d, 2H, *J* = 6.3, Ar-H), 7.40 (s, 2H, -SO_2_NH_2_), 3.88 (s, 3H, -OCH_3_), 3.45–3.28 (m, 4H, piperidine), 1.77–1.46 (m, 6H, piperidine):): ^13 ^C-NMR (DMSO-d_6_, 75 MHz, δ ppm): 169.1, 167.7, 165.1, 157.8, 143.6, 136.2, 130.6, 129.7, 120.1, 116.2, 56.8, 42.4, 25.1, 24.4;

##### 4-((4-((3,4-dichlorophenyl)amino)-6-(dimethylamino)-1,3,5-triazin-2-yl)amino) benzenesulfonamide (3 g)

Yield: 75%; Colour: white solid; m.p.: 254–257 °C; FT-IR (cm^−1^): 3353, 3270, 3262, 1600, 1502 (asymmetric), 1327, 1150 (symmetric) (S = O); ^1^H-NMR (DMSO-d_6_, 300 MHz, δ ppm): 8.04 (d, 2H, *J* = 6.6, Ar-H), 7.94 (d, 2H, *J* = 6.3, Ar-H), 7.58 (s, H, Ar-H), 7. 53 (d, 2H, *J* = 6.3, Ar-H), 7.42 (s, 2H, -SO_2_NH_2_), 3.08 (s, 6H, -CH_3_): ^13 ^C-NMR (DMSO-*d_6_*, 75 MHz, δ ppm): 169.8, 167.3, 165.6, 164.4, 157.5, 150.2, 141.7, 136.2, 130.3, 129.5, 126.6, 120.8, 118.3, 36.6;

##### 4-((4-((3,4-dichlorophenyl)amino)-6-morpholino-1,3,5-triazin-2-yl)amino) benzenesulfonamide (3 h)

Yield: 78%; Colour: white solid; m.p.: 199–201 °C; FT-IR (cm^−1^): 3329, 3211, 1625, 1490 (asymmetric), 1337, 1153 (symmetric) (S = O); ^1^H-NMR (DMSO-d_6_, 300 MHz, δ ppm): 8.06 (d, 2H, *J* = 6.6, Ar-H), 7.92 (d, 2H, *J* = 6.3, Ar-H), 7.57 (s, H, Ar-H), 7. 52 (d, 2H, *J* = 6.3, Ar-H), 7.44 (s, 2H, -SO_2_NH_2_), 3.81–3.46 (m, 8H, morpholine): ^13 ^C-NMR (DMSO-*d_6_*, 75 MHz, δ ppm): 169.4, 167.1, 165.8, 164.3, 157.2, 150.0, 141.9, 136.6, 130.8, 129.2, 126.5, 120.2, 118.6, 65.6, 42.2;

##### 4-((4-((3,4-dichlorophenyl)amino)-6-(piperidin-1-yl)-1,3,5-triazin-2-yl)amino) benzenesulfonamide (3i)

Yield: 81%; Colour: white solid; m.p.: 245–248 °C; FT-IR (cm^−1^): 3340, 3211, 2935, 2854, 1603, 1496 (asymmetric), 1361, 1155 (symmetric) (S = O); ^1^H-NMR (DMSO-d_6_, 300 MHz, δ ppm): 8.02 (d, 2H, *J* = 6.3, Ar-H), 7.89 (d, 2H, *J* = 6.0, Ar-H), 7.54 (s, H, Ar-H), 7. 50 (d, 2H, *J* = 6.0, Ar-H), 7.41 (s, 2H, -SO_2_NH_2_), 3.44–3.27 (m, 4H, piperidine), 1.72–1.41 (m, 6H, piperidine): ^13 ^C-NMR (DMSO-d_6_, 75 MHz, δ ppm): 168.2, 166.9, 165.2, 164.1, 157.8, 150.3, 141.5, 136.4, 130.3, 129.6, 126.2, 120.4, 118.7, 42.5, 25.1, 24.4;

##### 4-((4-(dimethylamino)-6-((3-nitrophenyl)amino)-1,3,5-triazin-2-yl)amino) benzenesulfonamide (3j)

Yield: 78%; Colour: light yellow solid; m.p.: 233–235 °C; FT-IR (cm^−1^): 3345, 3282, 3135, 1612, 1498 (asymmetric), 1345, 1158 (symmetric) (S = O); ^1^H-NMR (DMSO-*d_6_*, 300 MHz, δ ppm): 7.97 (d, 2H, *J* = 6.3, Ar-H), 7.87 (d, 2H, *J* = 6.3, Ar-H), 7.55 (s, H, Ar-H), 7. 51 (m, 3H, Ar-H), 7.42 (s, 2H, -SO_2_NH_2_), 3.05 (s, 6H, -CH_3_): ^13 ^C-NMR (DMSO-*d_6_*, 75 MHz, δ ppm): 169.6, 167.5, 164.9, 163.5, 157.2, 150.5, 142.1, 135.4, 130.7, 129.8, 125.4, 120.8, 117.2, 36.8;

##### 4-((4-morpholino-6-((3-nitrophenyl)amino)-1,3,5-triazin-2-yl)amino)benzenesulfonamide (3k)

Yield: 86%; Colour: light yellow solid; m.p.: 215–218 °C; FT-IR (cm^−1^): 3338, 3280, 3145, 1615, 1502 (asymmetric), 1348, 1159 (symmetric) (S = O); ^1^H-NMR (DMSO-*d_6_*, 300 MHz, δ ppm): 7.99 (d, 2H, *J* = 6.3, Ar-H), 7.85 (d, 2H, *J* = 6.3, Ar-H), 7.59 (s, H, Ar-H), 7. 52 (m, 3H, Ar-H), 7.44 (s, 2H, -SO_2_NH_2_), 3.83–3.48 (m, 8H, morpholine): ^13 ^C-NMR (DMSO-*d_6_*, 75 MHz, δ ppm): 169.0, 167.8, 164.5, 163.2, 157.8, 150.3, 142.4, 135.8, 130.1, 129.3, 125.6, 120.5, 117.8, 65.5, 42.4;

##### 4-((4-((3-nitrophenyl)amino)-6-(piperidin-1-yl)-1,3,5-triazin-2-yl)amino) benzenesulfonamide (3 l)

Yield: 84%; Colour: light yellow solid; m.p.: 181–184 °C; FT-IR (cm^−1^): 3341, 3215, 3125, 1602, 1502 (asymmetric), 1350, 1154 (symmetric) (S = O); ^1^H-NMR (DMSO-*d_6_*, 300 MHz, δ ppm): 7.96 (d, 2H, *J* = 6.0, Ar-H), 7.82 (d, 2H, *J* = 6.3, Ar-H), 7.58 (s, H, Ar-H), 7. 50 (m, 3H, Ar-H), 7.41 (s, 2H, -SO_2_NH_2_), 3.46–3.29 (m, 4H, piperidine), 1.74–1.45 (m, 6H, piperidine): ^13 ^C-NMR (DMSO-*d_6_*, 75 MHz, δ ppm): 168.8, 167.3, 164.6, 162.7, 157.4, 150.6, 142.1, 135.4, 130.7, 129.2, 125.1, 120.0, 117.2, 42.3, 25.0, 24.2;

### 2.2. Determination of the antioxidant, anticholinesterase and tyrosinase activity of benzenesulfonamides 2(a-d) and 3(a-l)

#### 2.2.1. DPPH radical scavenging assay

The DPPH (1,1-diphenyl-2-picrylhydrazyl) radical scavenging activity of the synthesised compounds was determined by spectrophotometric method based on the reduction of an ethanol solution of DPPH^23^^,^^24^. 2, 5, 10, 20 µL of 1 mM stock solution of each compound was completed to 40 µL with the DMSO and mixed with 160 µL of 0.1 mM of DPPH free radical solution. The mixture was led to stand for 30 min in the dark and the absorbance was then measured at 517 nm against a blank. Inhibition of free radical, DPPH, in percent (I %) was calculated as follows:

$$I\%=\left(A_{\text{control}}–A_{\text{sample}}\right)/A_{\text{control}}\times 100,$$

where A_control_ is the absorbance of the control reaction (containing all reagents except for the tested compounds), and A_sample_ is the absorbance of the test compounds. Tests were carried out in triplicate. BHA, BHT and α-Tocopherol were used as positive control^25–28^.

#### 2.2.2. ABTS cation radical decolorisation assay

The percent inhibition of decolorisation of ABTS (2,2`-azino-bis(3-ethylbenzothiazoline-6-sulfonic acid)) cation radical is obtained as a function of time and concentration, and evaluated by comparison with the BHT, BHA and α-Tocopherol compounds used as standard^29^^,^^30^. The tested compounds at different concentrations are added to each well and 160 µL of 7 mM ABTS solution is added. After 6 min at room temperature, the absorbances were measured at 734 nm. ABTS cation radical decolorisation activities were determined by

$$\%\text{Inhibition}=\left(A_{\text{control}}-A_{\text{sample}}\right)/A_{\text{control}}\times 100$$

where A is the absorbance. Tests were carried out in triplicate. BHA, BHT and α-Tocopherol were used as positive control.

#### 2.2.3. Metal chelating activity

The chelating ability of synthesised compounds was examined according to the method of Dinis et al.^31^ The tested compounds at different concentrations were added to each well and 4 µL of 2 mM ferrous (II) chloride was added. Then 8 µL of 5 mM ferrozine was added and the reaction was started. After 10 min at room temperature, the absorbance was measured at 562 nm against blank. The results were expressed as percentage of inhibition of the ferrozine-Fe^2+^ complex formation. The percentage inhibition of the ferrozine -Fe^2+^ complex formation was calculated as follows:

$$\text{Chelating}\text{ability}\left(\%\right)=\left(A_{\text{control}}-A_{\text{sample}}\right)/A_{\text{control}}\times 100,$$

where A is the absorbance. Tests were carried out in triplicate. EDTA was used as a positive control^32–34^.

#### 2.2.4. Anti-cholinesterase assay

The inhibitory effect of novel benzenesulfoanmides incorporating 1,3,5-triazine moieties **2(a-d)** and **3(a-l)** on AChE and BChE activities was determined according to the slightly modified spectrophotometric method of Ellman et al.^35^. All compounds were dissolved in DMSO to prepare stock solutions at 4 mM concentration. Aliquots of 150 µL of 100 mM sodium phosphate buffer (pH 8.0), 10 µL of sample solution and 20 µL AChE (or BChE) solution were mixed and incubated for 15 min at 25 °C, and DTNB (5,5’-Dithio-bis(2-nitro-benzoic)acid) (10 µL) is added. The reaction was then initiated by the addition of acetylthiocholine iodide (or butyrylthiocholine iodide) (10 µL). The final concentration of the tested compounds’ solution was 200 µM.

$$\%\text{Inhibition}=\left(A_{\text{control}}-A_{\text{sample}}\right)/A_{\text{control}}\times 100,$$

where A is the absorbance. Tests were carried out in triplicate. Galantamine was used as positive control.

#### 2.2.5. Anti-tyrosinase activity

Anti-tyrosinase activity of the compounds was performed according to the method designed by Hearing and Jimenez^36^. The inhibition of the diaphanous function of the compounds was evaluated with L-DOPA as substrate. Tyrosinase from mushroom (E.C. 1.14.18.1) (30 U, 28 nM) was dissolved in Na-phosphate buffer (pH = 6.8, 50 nM) and the compounds were added to the solution for pre-incubation at room temperature for ten minutes. After incubation, 0.5 mM L-DOPA was added to the mixture and the change in absorbance was measured at 475 nm at 37 °C. For the positive control, kojic acid was used as an inhibitor. The following formula was used to calculate the percentage of all enzyme inhibitions:

$$\text{Inhibition}\left(\%\right)=\left(A_{\text{control}}–A_{\text{sample}}\right)/A_{\text{control}}\times 100,$$

where *A* is absorbance.

### 2.3. Statistical analysis

The results of the antioxidant, anticholinesterase and tyrosinase activity assays are expressed as the mean ± SD of three parallel measurements. The statistical significance was estimated using a Student’s *t*-test, where *p* values < 0.05 were considered significant.

## 3. Result and discussion

### 3.1. Chemistry

**Scheme 1.**
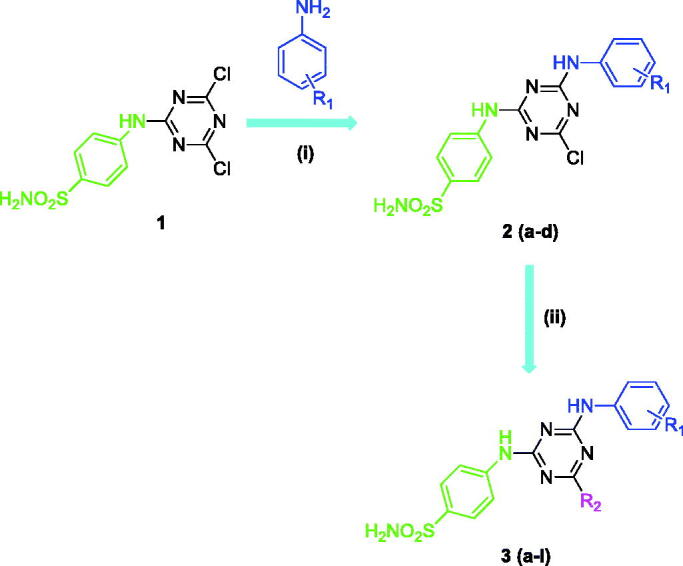
General synthetic route for the synthesis of benzenesulfonamides incorporating 1,3,5-triazine moieties. Reagents and conditions: (i) R_1_ (–4 F, –4MeO, –3,4diCl, and –3NO_2_), DMF, 0 to 5 °C, 1 h, then 30–40 °C, 8 h, (ii) R_2_H (dimethylamine, morpholine and piperidine), DMF, room temperature, 1 h, then 90 °C, 5 h.

The rationale for designing this novel benzenesulfonamides incorporating 1,3,5-triazine structural motifs presented in this work are based on our previous work which showed efficient carbonic anhydrase IX (tumour over-expressed isozyme) inhibition potency associated with such derivatives^5–12^. A number of structurally diverse benzenesulfonamides incorporating 1,3,5-triazine moieties were synthesised according to the general synthetic route depicted in Scheme 1. In order to generate chemical diversity, different substituted aromatic amines (-4 F, -4MeO, -3,4diCl and -3NO_2_ substituted anilines) were chosen and reacted at one side of the triazine moiety, whereas on the other side the derivatisation was achieved by using dimethlyamine, morpholine and piperidine functionalities.

The synthesis of benzenesulfonamides incorporating 1,3,5-triazine moieties **2(a-d)** and **3(a-l)** was carried out according to the procedure described in our previous papers^11^^,^^12^. Briefly, the starting key intermediate compound **1** was coupled with substituted aromatic anilines (-4 F, -4MeO, -3,4diCl and -3NO_2_), leading to formation of compounds **2(a-d)**. After that, the third chloride atom of the starting material 1,3,5-triazine (cyanuric chloride) was derivatised with dimethylamine, morpholine and piperidine to produce compounds **3(a-l)**. The structures of benzenesulfonamides incorporating 1,3,5-triazine moieties **2(a-d)** and **3(a-l)** were confirmed by using several analytical and spectral data (FT-IR, ^1^H-NMR, ^13 ^C-NMR, and melting points) as described in the experimental part.

### 3.2. Antioxidant activity

The benzenesulfonamides incorporating 1,3,5-triazine moieties were screened for their antioxidant activity by three methods, namely DPPH free radical scavenging, ABTS cation radical scavenging, and metal chelating activity. All of the compounds showed antioxidant activities in a dose-dependent manner and the results were summarised in Table 1, which demonstrates the IC_50_ values of the synthesised derivatives and standard compounds (BHA, BHT, α-tocopherol, and EDTA).

**Table 1.** Antioxidant activity of sulphonamides **2** and **3**.

			IC_50_ (µM)^a^		
			DPPH free radical	ABTS cation radical	Metal chelating
Comp.	R1	R2	Scavenging activity	Scavenging activity	Activity
**2a**	–4F	Cl	469.75 ± 1.17	>1000	488.29 ± 0.84
**2b**	–4MeO	Cl	500.97 ± 1.17	>1000	164.26 ± 0.68
**2c**	–3,4diCl	Cl	304.52 ± 1.38	>1000	109.63 ± 0.80
**2d**	–3NO_2_	Cl	443.26 ± 1.38	>1000	296.78 ± 0.52
**3a**	–4F	–N(Me)_2_	>1000	>1000	84.98 ± 1.14
**3b**	–4F		73.25 ± 0.52	>1000	148.03 ± 0.61
**3c**	–4F		>1000	>1000	338.90 ± 0.59
**3d**	–4MeO	–N(Me)_2_	102.65 ± 1.17	294.12 ± 1.20	337.51 ± 0.55
**3e**	–4MeO		>1000	>1000	84.32 ± 0.39
**3f**	–4MeO		>1000	408.44 ± 1.67	98.84 ± 0.90
**3g**	–3,4diCl	–N(Me)_2_	609.35 ± 0.98	>1000	139.15 ± 1.15
**3h**	–3,4diCl		60.18 ± 0.59	>1000	147.60 ± 0.82
**3i**	–3,4diCl		351.97 ± 1.33	>1000	99.10 ± 0.52
**3j**	–3NO_2_	–N(Me)_2_	58.59 ± 0.12	>1000	98.84 ± 0.90
**3k**	–3NO_2_		336.28 ± 1.43	481.21 ± 0.97	88.42 ± 0.75
**3l**	–3NO_2_		114.38 ± 0.60	>1000	115.46 ± 0.87
BHA^b^	–	–	61.72 ± 0.85	45.40 ± 1.08	–
BHT^b^	–	–	232.11 ± 3.01	26.54 ± 0.18	–
α-Tocopherol^b^	–	–	56.86 ± 0.77	34.12 ± 0.41	–
EDTA^b^	–	–	–	–	52.35 ± 1.15

^a^ IC_50_ values represent the means (standard deviation of three parallel measurements (*p* < 0.05).

^b^ Reference compounds.

The results revealed that benzenesulfonamides incorporating 1,3,5-triazine moieties **2(a-d)** and **3(a-l)** shows, in general, moderate DPPH radical scavenging and metal chelating activity, and low ABTS cation radical scavenging activity. Specifically, three compounds from the synthesised derivatives (**3 b**, **3 h** and **3j**) indicates high DPPH radical scavenging activity with IC_50_ values of 73.25, 60.18, and 58.59 µM, respectively. These compounds have better antioxidant activity than standards **BHA** (IC_50_: 61.72 µM) and **BHT** (IC_50_: 232.11 µM). On the other hand, compounds **3a**, **3c**, **3e**, and **3f** showed any activity with IC_50_ values of >1000 µM. Furthermore, the ABTS cation radical scavenging activity of the compounds was also assayed and compared with standards **BHT**, **BHA**, and **α-Tocopherol**. All compounds showed weak activity with IC_50_ values of <1000 µM, except the compounds **3d**, **3f** and **3k** exhibited moderate activity with IC_50_ values of 294.12, 408.44 and 481.21 µM, respectively (Table 1). The metal chelating activity of the synthesised compounds was also screened and compared with standard **EDTA**. None of the compounds showed better activity than standard. Moreover, several derivatives (**3a**, **3e** and **3k**) displayed close metal chelating activity to **EDTA** with IC_50_ values of 84.98, 84.32 and 88.42 µM, respectively. The remaining compounds were shown to have moderate metal chelating activity with IC_50_ values ranging from 98.84 to 488.29 µM.

In the present study, sulphonamides incorporating 1,3,5-triazine moieties **2(a-d)** and **3(a-l)** were also evaluated for their anticholinesterase (AChE and BChE) activities (Table 2). In general, all compounds showed better BChE inhibitory activity than AChE, except the compound **2 b**, which displayed more AChE inhibitory activity (% inhibition 96.37) than BChE (91.99). Compounds **2 b**, **3d** and **3 h** found to have most potent AChE inhibitors, having better % inhibition than standard drug galantamine, with % inhibition values of 96.37, 91.10 and 93.19, respectively. Some compounds from the series (**2a**, **2c**, **3a**, **3e**, **3f**, **3 g**, **3i**, **3j**, and **3 l**) showed any activity (NA) against AChE enzyme. The remaining compounds (**2d**, **3 b**, **3c**, and **3k**) showed low activity to AChE enzyme with % inhibition values ranging from 14.18 to 36.55. On the other hand, BChE enzyme was effectively inhibited by most of the synthesised compounds. Specifically, compounds **2 b**, **2d**, **3d**, **3f**, **3 h**, **3j**, and **3 l** showed >90% inhibition, which is higher than standard drug galantamine with a % inhibition value of 87.86. Among the remaining compounds, some of them (**2c**, **3 b**, **3c**, and **3i**) showed close % inhibition scale to standard drug with % inhibition values of 87.44, 88.76, 89.21, and 88.48, respectively. Only one non active compound (**3e**) was observed against BChE enzyme, in which this compound also inactive against AChE enzyme, too (Table 2). Interestingly, three compounds from the current series (**2 b**, **3d** and **3 h**) displayed higher activity against both enzyme (AChE and BChE) together than standard drug galantamine, which is a one of the most important findings of the current study. These three compounds gave promising anticholinesterase activity and might be improved and used as powerful cholinesterase inhibitors.

**Table 2.** Anti-cholinesterase and anti-tyrosinase activity of compounds **2** and **3**.

			Anticholinesterase activity^a^		
Comp.	R1	R2	AChE assay	BChE assay	Tyrosinase activity^a^
**2a**	–4F	Cl	NA	77.41 ± 1.19	38.96 ± 0.12
**2b**	–4MeO	Cl	96.37 ± 1.71	91.99 ± 0.60	14.21 ± 0.40
**2c**	–3,4diCl	Cl	NA	87.44 ± 1.46	33.44 ± 0.82
**2d**	–3NO_2_	Cl	22.38 ± 1.09	91.40 ± 1.48	NA
**3a**	–4F	–N(Me)_2_	NA	34.67 ± 0.83	13.50 ± 0.04
**3b**	–4F		14.18 ± 0.41	88.76 ± 0.97	15.34 ± 0.87
**3c**	–4F		36.55 ± 1.17	89.21 ± 1.75	6.29 ± 0.39
**3d**	–4MeO	–N(Me)_2_	91.10 ± 1.42	96.94 ± 1.61	27.76 ± 0.22
**3e**	–4MeO		NA	NA	30.37 ± 0.17
**3f**	–4MeO		NA	95.14 ± 1.17	86.35 ± 1.39
**3g**	–3,4diCl	–N(Me)_2_	NA	10.23 ± 0.70	58.49 ± 0.86
**3h**	–3,4diCl		93.19 ± 2.33	98.50 ± 1.63	24.13 ± 0.52
**3i**	–3,4diCl		NA	88.48 ± 1.29	23.21 ± 0.97
**3j**	–3NO_2_	–N(Me)_2_	NA	92.14 ± 1.02	77.30 ± 1.42
	–3NO_2_		28.42 ± 0.76	65.44 ± 1.19	18.40 ± 0.43
**3l**	–3NO_2_		NA	94.94 ± 0.24	58.59 ± 0.89
Galantamine^b^		–	84.20 ± 0.74	87.86 ± 0.24	–
Kojic Acid^b^		–	–	–	95.26 ± 0.23

^a^ % inhibition values at 200 µM.

^b^ Standard drugs. NA: not active.

Tyrosinase is a bi-functional copper containing enzyme which is widely distributed in different organisms such as animals, plants, and microorganisms^37^. This enzyme catalyses hydroxylation of monophenols to *o*-diphenols which generates melanin^37^^,^^38^. Tyrosinase inhibitors capable of inhibiting the biosynthesis of melanin are used for various applications in the food^39^, cosmetics^40^ and medicinal industries^37^^,^^38^. In the present study, tyrosinase enzyme inhibition activity was also evaluated and moderate activity was observed against this enzyme (Table 2). In general, none of the compounds showed better inhibition potency than standard Kojic acid, which has % inhibition value of 95.26. Two compounds showed high activity, **3f** and **3j**, with % inhibition values of 86.35 and 77.30, respectively. The remaining compounds indicated moderate activity depends on the substitution on compounds with % inhibition values ranging from 6.29 to 58.59. Only one compound (**2d**) was none active against this enzyme.

## 4. Conclusions

In the present study, we report a novel series of benzenesulfonamides incorporating 1,3,5-triazine moieties substituted with aromatic amine derivatives, dimethylamine, morpholine and piperidine. The novel compounds were investigated as an antioxidant and inhibitors of AChE, BChE and tyrosinase enzymes. The results revealed that benzenesulfonamides incorporating 1,3,5-triazine moieties **2(a-d)** and **3(a-l)** show, in general, moderate DPPH radical scavenging and metal chelating activity, and low ABTS cation radical scavenging activity. Compounds **2 b**, **3d** and **3 h** displayed great inhibition potency against AChE with % inhibition values of 96.37, 91.10 and 93.19, respectively. BChE also effectively inhibited by most of the synthesised compounds having >90% inhibition potency which is a one of the most important findings from the current work. Several lead compounds were also obtained against tyrosinase enzyme and they might be improved and used as effective inhibitors of these enzymes.
